# Multi Species Analyses Reveal Testicular T3 Metabolism and Signalling as a Target of Environmental Pesticides

**DOI:** 10.3390/cells10092187

**Published:** 2021-08-25

**Authors:** Valeria Nittoli, Marco Colella, Alfonsina Porciello, Carla Reale, Luca Roberto, Filomena Russo, Nicola A. Russo, Immacalata Porreca, Mario De Felice, Massimo Mallardo, Concetta Ambrosino

**Affiliations:** 1 Biogem, Istituto di Biologia e Genetica Molecolare, Via Camporeale, 83031 Ariano Irpino (AV), Italy; valeria.nittoli@biogem.it (V.N.); marco.colella@biogem.it (M.C.); alfonsina.porciello@biogem.it (A.P.); carla.reale@biogem.it (C.R.); luca.roberto@biogem.it (L.R.); filomena.russo@biogem.it (F.R.); nicola.russo@biogem.it (N.A.R.); immacolataporreca@gmail.com (I.P.); 2 Department of Science and Technology, University of Sannio, 82100 Benevento, Italy; 3 Laboratory of Pre-Clinical and Translational Research, IRCCS, Referral Cancer Center of Basilicata, 85028 Potenza, Italy; 4 Department of Molecular Medicine and Medical Biotechnologies, University of Naples “Federico II”, 59100 Naples, Italy; mario.defelice@unina.it; 5 IEOS-CNR, 80131 Naples, Italy

**Keywords:** pesticides, T3 signalling and metabolism, cross-species analysis, testis, fertility

## Abstract

Thyroid hormones (THs) regulate many biological processes in vertebrates, including reproduction. Testicular somatic and germ cells are equipped with the arrays of enzymes (deiodinases), transporters, and receptors necessary to locally maintain the optimal level of THs and their signalling, needed for their functions and spermatogenesis. Pesticides, as chlorpyrifos (CPF) and ethylene thiourea (ETU), impair the function of thyroid and testis, affecting male fertility. However, their ability to disarrange testicular T3 (t-T3) metabolism and signalling is poorly considered. Here, a multi-species analysis involving zebrafish and mouse suggests the damage of t-T3 metabolism and signalling as a mechanism of gonadic toxicity of low-doses CPF and ETU. Indeed, the developmental exposure to both compounds reduces *Dio2* transcript in both models, as well as in ex-vivo cultures of murine seminiferous tubules, and it is linked to alteration of steroidogenesis and germ cell differentiation. A major impact on spermatogonia was confirmed molecularly by the expression of their markers and morphologically evidenced in zebrafish. The results reveal that in the adopted models, exposure to both pesticides alters the t-T3 metabolism and signalling, affecting the reproductive capability. Our data, together with previous reports suggest zebrafish as an evaluable model in assessing the action of compounds impairing locally T3 signalling.

## 1. Introduction

Thyroid hormones (thyroxine, T4; triiodothyronine, T3) are major regulators of development, differentiation, and metabolism in multiple mammalian tissues. In vertebrates, THs activity is evolutionary conserved and depends on both circulating and tissue/organ levels established locally by a cell/tissue specific array of metabolizing enzymes (deiodinases), transporters, and receptors [1]. Despite testis having been considered a thyroid unresponsive organ for a long time, the presence of deiodinases and receptors, identified in developing and adult testicular somatic and germ cells, have suggested an important role for THs in their function [2,3]. In vivo and in vitro evidence have explained that T3 modulates Sertoli cells (SCs) proliferation and differentiation during testis development and participates to the assembly of the blood–testis barrier (BTB) in rodents [3,4,5]. In vitro studies have demonstrated that T3 inhibited the follicle stimulating hormone (FSH)-dependent mitosis of SCs [4,6] and the expression of immature SCs markers such as Anti-Müllerian hormone (AMH) [7,8]. Furthermore, an in vivo study conducted in mice treated with propylthiouracil (PTU) or T3 showed a significant increase of total number of SCs per testis in PTU-treated mice in comparison to the controls whereas the opposite occurred in T3-treated mice [9]. Developmental hypothyroidism affects spermatogenesis by reducing the differentiation of spermatogonia [10] and it may result in increased sperm production in adulthood, due to a larger reserve of undifferentiated germ cells [9]. Moreover, T3 is necessary to initiate the differentiation of mesenchymal cells into Leydig progenitor cells and to promote the development of Leydig cells (LCs) in concert with other hormones as luteinizing hormone (LH) and IGF-I [11,12,13]. This effect is associated with decreased expression of steroidogenic genes, such as *Star* [14,15]. Although THs are generally accepted as important regulators of the testicular development in mammals, their role is not very clear in the adult testis [16]. Recently, the altered thyroid status in males has been associated with abnormal spermatogenesis, reduced sexual activity, and impeded fertility [17]. Indeed, hypothyroidism might decrease the levels of serum testosterone and alter sperm morphology [18,19]. Vice versa hyperthyroidism is commonly associated with lower sperm motility in humans [20].

Due to the close degree of similarity of regulation of the reproductive systems between mammals and zebrafish, this model is increasingly used in reproductive sciences [21]. Male zebrafish have paired testes with tubule organizations. Within each tubule, the walls are lined by SCs, mainly supporting the testicular morphogenesis and spermatogenesis. LCs, detected in the interstitial spaces, act as primary androgen producers [22,23]. One distinct spermatogenesis pattern observed in zebrafish is the presence of spermatogenic cyst: cytoplasmic extensions of SCs form cysts by enveloping a synchronously developing germ cell clone derived from a single spermatogonial cell. This is defined by the type A undifferentiated spermatogonia (A_und_) [24,25]. More recently, using an ex vivo approach, it has been showed that T3 increased mitotic index of both spermatogonia (A_und_) and SCs in zebrafish testis. It also stimulated the accumulation of type A differentiated spermatogonia (A_diff_) by increasing their proliferation and reducing their development into type B spermatogonia [24,26]. T3 action is exerted by modulating the expression of selected insulin-like growth factor-binding protein, Igfbps, such as Igfbp1a. These proteins are considered relevant for modulation of insulin growth factors (IGFs) in zebrafish testis [27,28,29]. THs may also regulate the functions of LCs in zebrafish testis, being their steroidogenic capacity increased by T3 [24]. However, the pathway to androgen production is somehow different in zebrafish compared to mammals [30,31,32]. Indeed, the principal active androgen is 11-ketotestosterone (11-KT) in zebrafish, produced via multiple pathways involving three key enzymes: 11β-hydroxylase (Cyp11c1), 11-β-dehydrogenase isozyme 2 (Hsd11b2), and 17β-Hydroxysteroid dehydrogenase 3 (Hsd17b3) [30,31].

THs and testicular functions are controlled by environmental stressors, including pesticides. Environmental pesticides are widely used due to the farming intensification [33]. Pesticides, such as organophosphate compounds (Chlorpyrifos, CPF) and carbamates, (ethylene thiourea, ETU, the main metabolite of mancozeb), are considered Endocrine Disrupting Chemicals (EDCs), able to interfere with natural hormones, even at low doses, and to affect the normal development and function of multiple organs [34,35]. Experimental data provide evidence that long-term exposure to ETU and/or CPF impair levels of THs in rodents [36,37,38]. Recently, we have investigated the effects of embryonic and long-life exposure to low-dose of ETU and CPF in zebrafish, revealing their ability to alter the hepatic metabolism and signalling of T3 [39]. Considering that in vivo and in vitro studies have provided evidences that both CPF and ETU can also impair male fertility [40,41], we investigated if testicular T3 (t-T3) metabolism and signalling are targeted by both pesticides and if this is related to the impairment of the testicular function. We used a model of developmental and long-life exposure to low doses of ETU and CPF on adult testis in zebrafish and mice and of ex-vivo cultures of seminiferous tubules.

## 2. Materials and Methods

### 2.1. Mouse Treatment

Mice were housed in a Biogem Animal facility under standard facility conditions and received water and standard diet (4RF21, Mucedola) “ad libitum”. All animal experiments were performed in accordance with the European Council Directive 2010/63/EU following the rules of the D.Lvo 116/92 (ID number 25-10), and the procedures were approved by the Ethical committee named CESA (Committee for the Ethics of the Experimentations on Animals) of the Biogem Institute. The number of the mice enrolled in the study was established executing a G-Power analysis, required in preparing the documents to obtain the authorization from the Italian Ministry of Health, that fix the parameters to use (α = 0.01; 1-β = 0.85; δ = 4.12). Doses of both compounds were chosen according to previously published data [42,43]. CD1 dams (outbred strain, 8 mice/treatment group) were exposed, 7 days before the mating, to pesticides dosed at 10 mg/kg/day, 1 mg/kg/day, 0.1 mg/kg/day, administrating ETU by drinking water (59, 5.9, and 0.59 mg/L), and CPF by food at 44, 4.4, and 0.44 mg/kg (Mucedola) until the weaning. The offspring were exposed through the mothers from gestational day 0 (GD 0) until the weaning, and then, they were directly exposed. The dose of both pesticides was chosen up to published reports in which not systemic effects of the exposure were reported [36] and are consistent with the NOAEL for CPF (0.3 mg/kg/die) and its relevant long-term NOAEL (0.9 mg/kg/die, 18 months) reported in the last statement of EFSA for CPF (approved 31 July 2019). Similarly, ETU relevant long-term NOAEL is 13 mg/kg/die (18 months). Finally, the relevant reproductive NOAEL corresponding to 5 mg/kg/die and 70 mg/kg/die for CPF and ETU, respectively [44,45]. Animals were sacrificed at 6 months, for blood and organs collection, by carbon dioxide inhalation.

### 2.2. Zebrafish Husbandry and Treatment

Adult fishes (AB line) were maintained according to standard procedures on a 14-h light/10-h dark lighting cycle at 28 °C. Animal experiments were performed in accordance with the European Council Directive 2010/63/EU and procedures were approved by the Italian Minister of Health (IMH, ID number 78-17). The number of the animals enrolled in the study was established executing a G-Power analysis, required in preparing the documents to obtain the authorization from the Italian Ministry of Health that fix the parameters to use (α = 0.01; 1-β = 0.85; δ = 4.12). Chlorpyrifos (CPF) and ethylene thiourea (ETU), purchased from Greyhound Chromatography and Allied Chemicals and Sigma Aldrich respectively, were stored as recommended. Doses of both compounds were chosen according to literature [46,47,48,49]. As previously described [39], zebrafish eggs at 6 hpf (hours post-fertilization), were randomly collected and placed in separate glass Petri dishes in 100 mL of fish water containing CPF (30 and 300 nM), ETU (100 μM) and were maintained in an incubator at 28 °C. The exposure doses were established referring to previously published results that were also experimentally verified by us during the setting up of our models [39]. They are environmentally relevant for the areas interested by a massive agricultural activity, as India and China. Indeed, they are 10 times higher than the ones reported by US EPA (1998) in its survey of chlorpyrifos in surface waters.

The exposure solutions were renewing daily and the animals were examined under a dissecting microscope for the morphological evaluation. Those exhibiting arrested development or malformations were discarded. At 10–15 days post fertilization (dpf), larvae were transferred in Stand Alone (Tescniplast), adapted for toxicology treatment, and the exposure were continued until the adulthood (180 dpf).

### 2.3. Analysis of Adult Seminiferous Tubules Cultured in Hanging Drop Condition

The hanging drop approach was chosen to support the culture of testis tubules fragments, as previously described [50,51], required to assess ex-vivo the effects of the exposure to pesticides and T3. Testes from C57/BL6 mice, housed in the Biogem Animal mice facility (AMF) were used (ID Authorization: 133-20_ 7F782.67). Briefly, dissected testes were placed in media, decapsulated and tubules manually dissociated and cut. Tubule fragments were carefully transferred and cultured in 30-μL hanging drops in DMEM F12 medium containing: 1× Penicillin/Streptomycin (P4333, Sigma-Aldrich, Saint Louis, MO, USA), insulin (10 μg/mL), transferrin (5.5 μg/mL), and supplemented with 10% charcoal stripped fetal bovine serum (F6765, Sigma-Aldrich) at 32 °C in 5% CO_2_/95% air. One tubule fragment was cultured in each hanging drop and each Petri dish contained approximately 30 drops. T3 (100 nM), CPF, and ETU (10 nM) was added to the culture medium and incubation was continued for 7 days. Culture media was changed every 2 days. The experiment was repeated in triplicate.

### 2.4. Gas Chromatography Mass Spectrometry (GC-MS) Analysis

Serum samples (N°5/group) for testosterone and estradiol quantifications were submitted to liquid-liquid extraction procedure followed by GC-MS analysis of the hexane supernatant as already reported [52].

### 2.5. RT-qPCR Analysis

Total RNA, from mouse and zebrafish testes (N°3/group) and from pooled mouse tubules, was isolated with TRIzol reagent (Invitrogen) according to the manufacturer’s instructions.

Reverse transcription (RT) and qPCR were accomplished using the QuantiTect Reverse Transcription Kit (Qiagen) and Fast SYBR Green Master Mix (Applied Biosystems with Applied Biosystem QuantStudio 7 Flex System), respectively. Primer sequences are listed in the Supplemental Material (see Supplementary Table S1). RTqPCR analyses were preceded by the determination of the levels of 3 different internal controls *(β-actin*, *Gapdh*, and *18S* for mouse, and *β-actin*, *elf1a*, and *tubaI* for zebrafish), in the different exposure conditions in order to verify their stability in the different exposure conditions. Data were normalized by the level of internal control *β-actin* expression in each sample because it was the more stable among them [53,54]. In order to avoid genomic contamination, we usually design primer sequences spanning an exon-exon junction. Experiments were performed in triplicates. The 2^−∆∆Ct^ method was used to calculate relative expression changes.

### 2.6. Measurement of Thyroid and Sexual Hormones in Tissue Homogenate

Free THs (fT4 and fT3), Testosterone, and 17-βEstradiol (E2) levels were measured on homogenates of adult testes (*n* = 3/group), using an enzyme-linked immunosorbent assay (ELISA) according to published protocol with minor changes [39]. Briefly, samples were homogenized in RIPA buffer: 50 mM Tris (pH 7.4), 150 mM NaCl, 0.1% SDS, 0.5% Na-deoxycholate, Nonidet P-40, protease and phosphatase inhibitor mixture (Sigma), using the tissue lyser instrument. After centrifugation (10 min, 5000× *g* at 4 °C), the supernatants were collected and stored at −80 °C until hormones measurement by the ELISA kit (Diametra kit: Estradiol, DKO003, sensitivity 8.7 pg/mL; Testosterone, DKO002, sensitivity 0.07 ng/mL; Free T3, DKO037, sensitivity 0.05 pg/mL; Free T4, DKO038, sensitivity 0.05 ng/dL) or used for Western blotting analyses.

### 2.7. Western Blotting Analysis

To prepare proteins, frozen testis (*n* = 3/group) were lysed by tissue lyser in RIPA buffer (50 mM Tris (pH 7.4), 150 mM NaCl, 0.1% SDS, 0.5% Na-deoxycholate, Nonidet P-40, protease and phosphatase inhibitor mixture (Sigma)) according to published protocol [39]. The following antibodies were used to detect both mouse and zebrafish proteins: anti-Estrogen Receptor alpha (ERα, Santa Cruz, sc-8002, 1:1000), anti-Deiodinasi 3 (DIO3, Novusbio NBP1-05767, 1:1000), and anti-CYP19A1 (CYP19A1, Elabscience, E-AB-31086, 1:1000). B-actin (Cell Signalling, 1:3000) was used to normalize data.

The secondary antibodies used were anti-rabbit (G21234, 1:2000) and anti-mouse (G21D40, 1:2000) (Life Technologies).

### 2.8. Hematoxylin and Eosin Stining and Immuno-Histochemistry

For microscopy, zebrafish testes were fixed in formalin and embedded in paraffin. Sections were stained with hematoxylin and eosin (Sigma-Aldrich) according to the manufacturer’s instructions.

Immunostaining was performed on 5 μm tissue sections from 3 samples/group of testis. Briefly, sections were deparaffinized and rehydrated ((Xylene 2× 3 min), (100% Ethanol 2× 3 min), (95% Ethanol 2× 3 min), (70% Ethanol 2× 3 min), Water)). The antigen retrieval was conducted by boiling for 20 min in Sodium Citrate Buffer (10 mM Sodium Citrate, 0.05% Tween 20, pH 6.0) and anti-Erα (1:200) was incubated O/N at 4 °C in PBS. Alexa-Fluor conjugate secondary antibody was used (1:1000) for 1 h at room temperature. After several washes, sections were mounted on coverslips with glycerol. DAPI staining to visualize nuclei was used (1:2000 for 10 min). Zebrafish samples were imaged on a Zeiss Axioplan 2 microscope, with 20× (for immunofluorescence) and 63× objectives (for Hematoxylin and Eosin staining).

### 2.9. Statistical Analysis

Statistical analyses were performed using the Prism 5.0 software (GraphPad Software, La Jolla, CA, USA). Student’s *t*-test and one-way ANOVA (post hoc test:Dunnett’s) for multiple comparisons were used. Probability *p*-values below 0.05 were considered significant and indicated respectively with the symbols (#) and (*). Unless otherwise indicated, three independent experiments were considered for in vitro data and a minimum of three animals for in vivo experiments. Fold change (FC) values were calculated as the ratio between average results in treated and control samples. The results are expressed as the mean ± standard deviation of independent experiments and animals for in vitro and the in vivo data, respectively.

## 3. Results

### 3.1. Developmental and Long-Term Exposure to Pesticide Affects Steroidogenesis and Metabolism of THs in Mouse

The effect on testes of low-dose CPF and ETU was evaluated in mice exposed since the conception and long-life (Supplementary Figure S1A). Specifically, we used the following: (a) low dose CPF (0.1 mg/kg/day, CPF-L, from now on), (b) medium dose CPF (1 mg/kg/day, CPF-M, from now on), (c) high dose CPF (10 mg/kg/day, CPF-H, from now on), (d) low dose ETU (0.1 mg/kg/day, ETU-L, from now on), (e) medium dose ETU (1 mg/kg/day, ETU-M, from now on), and (f) high dose ETU (10 mg/kg/day, ETU-H, from now on). These experimental settings were considered relevant to humans not professionally exposed in terms of exposure window and dose. Indeed, both compounds have been retrieved in the amniotic fluid [55] and cord blood [56,57] of general population, confirming that the exposure is started during the foetal life and continued during the all life. The dose used to medicate the food were chosen considering published reports and in line with the indication released by EFSA for both, as detailed in Material and Methods (M&M, from now on). 

**Figure 1:**
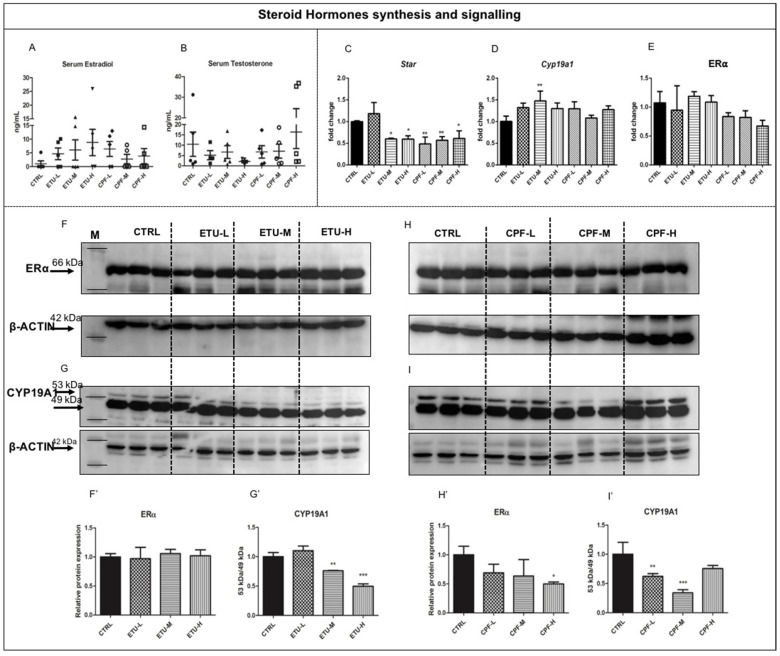
Developmental exposure to different dose of ETU and CPF effects steroid hormones biosynthesis and E2 signaling in mouse testis. (**A**,**B**) Serum free testosterone and 17-β estradiol levels were detected by GC/MS assay in mouse exposed to pesticides. (**C**–**E**) Levels of *Star*, *Cyp19a1*, and *Erα* transcript assessed by RT-qPCR analysis, performed on three biological samples (*n* = 3 testis/group). Data are reported as fold change values calculated as a ratio between average relative gene expression in treated and control testes, after normalization on *β-actin* mRNA. Mean and standard deviation are reported. (**F**,**F’**,**G**,**G’**) Western blot analysis showing the levels of ERα and CYP19A1 proteins following ETU treatment. Three mice were analyzed within the same group. Two isoforms were detected for CYP19A1 of 53kDa and 49kDa. Normalization was done detecting B-ACTIN. (**H**,**H’**,**I**,**I’**) Western blot analysis showing the levels of ERα and CYP19A1 proteins following CPF treatment, conducted as described above. Data analysis was conducted as described in M&M. One-way ANOVA (post hoc test: Dunnett’s) was used. * *p* < 0.05, ** *p* < 0.01, *** *p* < 0.001.

The levels of plasma free 17-β estradiol (E2, from now on; Figure 1A) and testosterone (Figure 1B) were determined by GC/MS, as detailed in M&M section. Although not statistically significant, the data evidence a trend towards the decrease of the testosterone levels in ETU-treated groups, particularly evident in ETU-H, and a trend toward the increase in CPF-H treated mice comparing both to not exposed males (CTRL, from now on, Figure 1B). The level of serum E2, vice versa, showed a trend towards the increase in all treated groups, especially in ETU-H sample (Figure 1A) vs. CTRL. We corroborated these data determining the levels of transcripts encoding for the enzymes involved in sex steroid hormones synthesis in testis, by RT-qPCR technique. We found that *Star* mRNA was strongly reduced in ETU and CPF treated testes (CPF- and ETU-testes, from now on) vs. CTRL, with the exception for ETU-L samples (Figure 1C). Interestingly, we observed an increase of *Cyp19a1* gene expression in ETU-M and a trend towards the increase in the other exposed groups, implying an increased conversion of testosterone to E2 (Figure 1D) and in agreement with Kumar P. et al. (2009). However, when we looked at CYP19A1 protein (Figure 1G,G’,I,I’), we detected a reduction of its level in all the exposed groups, although statistically relevant only for ETU-M and ETU-H and CPF-L and CPF-M samples (Figure 1G’,I’). Then, we analyzed also the testicular levels of Estrogen Receptor alpha transcript (*Erα*, Figure 1E) and protein (Figure 1F,F’,H,H’), and found a reduction of the last in CPF-H males.

**Figure 2:**
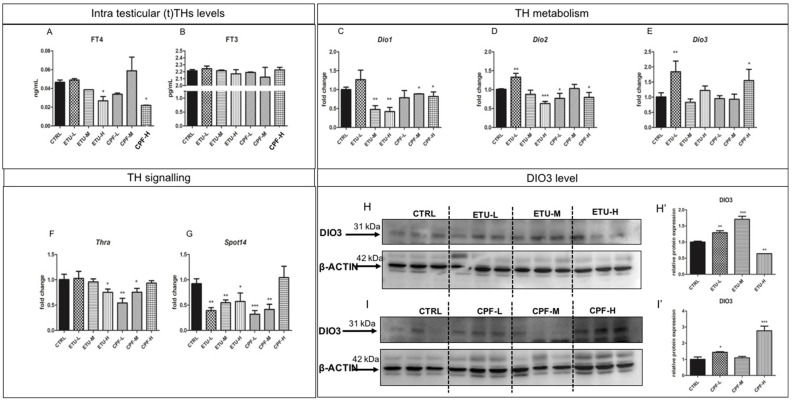
Developmental exposure to CPF and ETU modulates THs levels and metabolism in mouse testis. (**A**,**B**) Testicular (t)FT4 and (t)FT3 levels were measured by ELISA in adult testes from CTRL and exposed males (N° = 3 testis/group), as described in M&M section. (**C**–**G**) Levels of the mRNAs of enzymes involved in THs metabolism (*Dio1*, *Dio2*, *Dio3)* and signaling (*Thra*, *Spot14*) in testes of mice exposed to pesticides and CTRL. RTqPCR tests were performed on three biological samples (*n* = 3 testis/group). Data are reported as fold change values calculated as a ratio between average relative gene expression in treated and control testes, after normalization on *β-actin* mRNA. (**H**,**H’**,**I**,**I’**) Western blot analysis showing the level of DIO3 protein following ETU and CPF treatment (N°3 testis/group). Data were obtained normalizing using B-ACTIN. Data analysis was conducted as described in M&M. One-way ANOVA (post hoc test: Dunnett’s) was used. * *p* < 0.05, ** *p* < 0.01, *** *p* < 0.001. Mean and standard deviation are reported.

*Cyp19a1* and *Erα* are reported as targets of T3 in other models [58]. Since none major impairment of circulating T3 was detected in the exposed mice (Giacco et al., manuscript in preparation), we analyzed the effect of both pesticides on testicular TH (tTHs, from now on) metabolism and signalling. Firstly, we assessed the intra-testicular levels of free T4 (tfT4) and T3 (tfT3) by ELISA (Figure 2A,B) on tissue homogenates. Interestingly, we found a statistically significant reduction of tfT4 level in both ETU-H and CPF-H groups (Figure 2A) while the level of tfT3 was not affected (Figure 2B). Then, we evaluated the expression levels of deiodinases and of TH receptor alpha (*Thra*), shown to be expressed in adult testes [59]. As shown in Figure 2C, *Dio1* gene, converting T4 in T3, was reduced at the medium and high dose of CPF and ETU. A statistically significant reduction was reported in CPF-L and CPF-H testes and in ETU-H testes for *Dio2* mRNA (Figure 2D), encoding for the most abundant enzyme exerting the same activity (Supplementary Figure S2), whereas its increase was detected for ETU-L (Figure 2D). *Dio3* mRNA, the main T3 degrading enzyme, was increased in ETU-L and in CPF-H testes vs. CTRL (Figure 2E). This was surprising, since *Dio3* mRNA changes are detected mainly during the foetal/neonatal life. Then, we also evaluated the levels of DIO3 protein in the testes. As detailed in Figure 2H,I, we found a significant reduction of DIO3 protein level in ETU-H, while in the other groups it was found increased (Figure 2H,H’,I,I’). Finally, we assessed the effects on the expression of *Thra* transcript, retrieving its decrease in both ETU-H, CPF-L, and CPF-M groups compared with CTRL (Figure 2F). To describe the effects of the local damage of T3 signalling, we analyzed the expression of *Spot14*, a T3-responsive gene. We observed that almost all samples showed a reduced level of *Spot14* mRNA, with exception of the CPF-H group (Figure 2G). Overall, the data suggest that pesticides alter testicular E2 and THs synthesis and signalling.

### 3.2. Pesticides Directly Affect the Levels of E2 and TH Metabolism/Signalling Transcripts and of Markers of Testicular Somatic and Germ Cells in Ex-Vivo Cultures of Seminiferous Tubules

**Figure 3:**
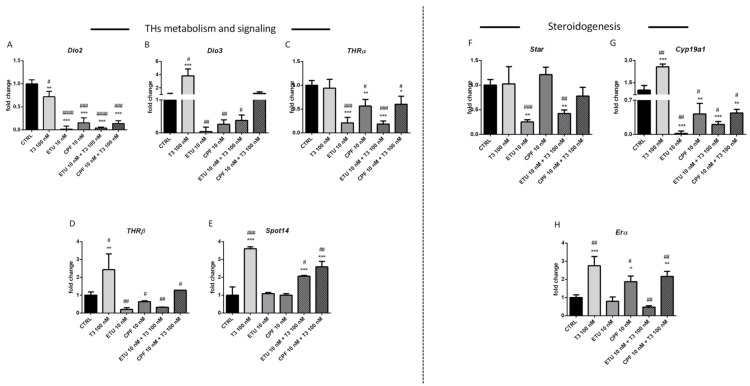

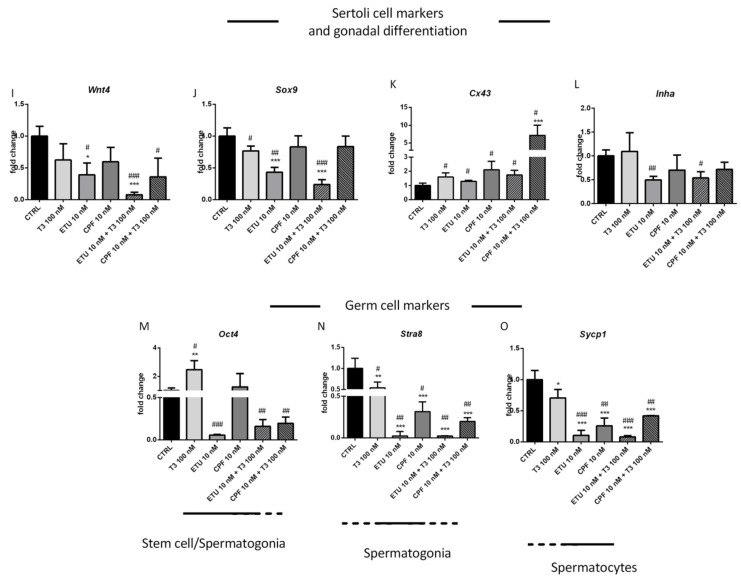
Exposure to T3, CPF, ETU, and their mixture modulate THs metabolism and alter both steroidogenesis and spermatogenesis in ex vivo seminiferous mouse tubule culture. (**A**–**E**) Levels of the mRNAs of enzymes involved in THs metabolism (*Dio1*, *Dio2*, *Dio3)* and signaling (*Thra*, *Spot14*); (**F**–**H**) in steroidogenesis and (**I**–**O**) in Sertoli and germ cells differentiation and proliferation in isolated seminiferous tubules exposed to T3, pesticides or a mixture of both. RT-qPCR tests were performed on pooled tubules fragments (*n* = 3 independent experiments). Data are reported as fold change values calculated as a ratio between average relative gene expression in treated and control testes, after normalization on *β-actin* mRNA. Mean and standard deviation are reported. Data analysis was conducted as described in M&M section. One-way ANOVA (post hoc test: Dunnett’s) was indicated with * *p* < 0.05, ** *p* < 0.01, *** *p* < 0.001. Student’s *t*-test was indicated with # *p* < 0.05, ## *p* < 0.01, ### *p* < 0.001, #### *p* < 0.0001.

As said, T3 signalling modulates the testicular *Cyp19a1* and *ERα* expression. In order to evaluate if their modulation is directly regulated by CPF and ETU or by impairment of tT3 promoted by both, we assessed their effects on ex-vivo cultures of seminiferous tubules. To this aim, we adopted the hanging drop approach, since already used to investigate the tubular response to other stimuli. The hanging drop cultures were set exposing them to stimuli for one week, in order to simulate chronic exposure [37]. Firstly, we assessed the effects of the CPF and ETU exposure on the levels of the transcripts of genes modulating TH metabolism and signalling (Figure 3). ETU (10 nM) and CPF (10 nM) were more effective of T3 (100 nM) in reducing the levels of *Dio2 (*Figure 3A). Moreover, *Dio3* transcript was reduced by pesticides and positively regulated by T3 (Figure 3B). The effect was not majorly counteracted by T3 co-administration for *Dio2*, whereas *Dio3* mRNA returned at control levels in the case of T3 and CPF co-treatment. Noteworthy, ETU significantly opposed to the induction of *Dio3* mRNA promoted by T3. Regarding *THR*s expression, we found that both compounds down-regulated their expressions (Figure 3C,D). In particular, none major impact on the activity of pesticides was evidenced on *THRα* mRNA when T3 was added (Figure 3C). Instead condition T3 restored the levels of *THRβ* mRNA when co-exposure conditions were used (Figure 3D). Although not affecting directly the level of *Spot14* mRNA, both compounds partially antagonize the activity of T3 (Figure 3E).

Regarding steroidogenesis, the levels of *Cyp19a1* and *Erα* mRNAs were increased by T3 (100 nM) treatment (Figure 3G,H). *Cyp19a1* mRNA levels were strongly reduced in tubules exposed to ETU 10 nM (Figure 3G). Such an effect was partially counteracted by T3 co-treatment. In the same samples none significant modulation of *Erα* mRNA was detected (Figure 3H). CPF treatment (10 nM) resulted in the increase of *Erα* transcript in tubules and co-treatment slightly increased it (Figure 3H). *Cyp19a1* mRNA was strongly reduced by CPF (10 nM) and the effect was not blunted by T3. Regarding *Star* transcript (Figure 3F), it was reduced by ETU and T3 partially reversed such effect.

Since spermiogenesis is a complex process tightly regulated by different hormonal axis in vivo, we took advantage from ex-vivo cultures of the seminiferous tubules to assess the effects of both compounds on the expression of specific markers of Sertoli cells (SCs, from now on) and germ cells. In particular, we assessed the levels of SRY-box9 mRNA (*Sox9*), a marker of SCs, as well as of Inhibin A (*Inha*) that promotes SCs proliferation in mice. We also looked at *Wnt4* since this pathway has been involved in male sexual development [60]. Finally, we tested the level of Connexin 43 (*Cx43*) transcript, the most abundant gap junction protein in testicular cells [61] controlling SC proliferation induced by THs and also positively regulated by T3 signalling [62]. We found that treatment with ETU (10 nM) decreased both *Wnt4* and *Sox9* transcripts (Figure 3I,J), while CPF (10 nM) treatment had an opposite effect. Both positively regulated the levels of *Cx43* mRNA as T3 (100 nM) (Figure 3K). Interestingly, the co-administration of CPF and T3 produced a strong up-regulation of *Cx43* mRNA level (Figure 3K). *Inha* was inhibited by ETU also when T3 was added (Figure 3L). Subsequently, we looked at germ cells markers evidencing that *Oct4* (marker of stem germ cells) was positively regulated by T3, whereas ETU had a strong negative impact (Figure 3M). Both compounds antagonized the positive effect of T3 (Figure 3M). *Stra8* transcript, marker of differentiated spermatogonia, was always reduced and the co-administration of T3 enforced the effect (Figure 3N). Finally, both pesticides and T3 also reduced the transcript of the synaptonemal complex protein 1 (*Sypc1*, Figure 3O), a spermatocyte marker. Overall, the data evidenced that CPF and ETU can directly modulate THs and E2 metabolism and signalling and such activity can be influenced by the presence of T3. Furthermore, the data evidence also a direct role on the spermiogenesis.

### 3.3. Pesticides Downregulate Key Genes Involved in Testis Development and Spermatogenesis

**Figure 4:**
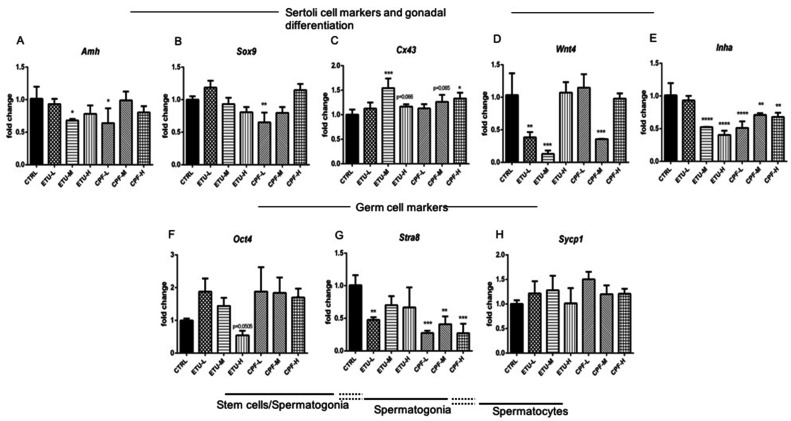
Developmental exposure to CPF and ETU modulates murine regulatory markers of testis function and gamete maturation. RT-qPCR analyses of markers of Sertoli cells (**A**–**E**) and of germ cells (**F**–**H**) from mouse adult testes exposed to pesticides since conception until adulthood and control. RT-qPCR analyses were performed on three biological samples (N° = 3 testis/group). Data analysis was conducted as described in M&M section. Data are reported as fold change values calculated as a ratio between average relative gene expression in treated and control, after normalization on *β-actin* mRNA. Mean and standard deviation are reported. Significant difference from the control group is indicated by * *p* < 0.05, ** *p* < 0.01. One-way ANOVA (post hoc test: Dunnett’s) was used.

The results obtained in the ex-vivo cultures of seminiferous tubules suggested that CPF, ETU, and also T3 affect differently the spermatogenesis. Thus, we investigated the effect of both pesticides on this process in exposed mice, by RT-qPCR method. In treated animals, the level of Anti-Mullerian hormone (*Amh*) that, together with *Sox9*, is produced and released by SCs, was reduced in ETU-M and CPF-L testes (Figure 4A). *Sox9* transcript decreased in CPF-L and showed a trend toward the reduction in ETU-H and CPF-M vs. CTRL testes, whereas its induction was evidenced in ETU-L and CPF-H treatment groups (Figure 4B). *Cx43* transcript showed a trend towards the upregulation, reaching the statistical significance in ETU-M and CPF-H testes compared to CTRL (Figure 4C). *Wnt4* mRNA was reduced in ETU-L, ETU-M and in CPF-M samples (Figure 4D), while *Inha* mRNA was inhibited in all exposed groups, with the exception of ETU-L (Figure 4E).

Then, we tested the transcription levels of markers of various stage of male germ cells differentiation. The data revealed a different regulation of each testicular germ cell sub-population. Indeed, mRNA levels of the stem germ cells transcription factor *Oct4*, and of the spermatocyte marker *Sypc1*, were increased in all CPF-testes and in ETU-L and ETU–M testes (Figure 4F,H) while *Stra8* transcript, marker of spermatogonia, decreased in statistically significant manner only in CPF samples and ETU-L (Figure 4G). Overall, the results suggest that developmental and long-term exposure to different doses of CPF and ETU alters SCs activity and function, and testicular cells population also in vivo.

### 3.4. Developmental and Long-Life Exposure to Pesticides Impair Testicular Thyroid Hormones Metabolism and Signalling in Zebrafish

Zebrafish has been proposed as a valid model to investigate mechanisms of human disease and to evaluate developmental effects of the environmental toxicants [21]. To corroborate the suggestion that t-T3 metabolism and signalling is impaired by developmental and long-life exposure to environmental pesticides, we investigated both in zebrafish testes collected during a study aimed to characterize the same issue in liver [39]. Animals were exposed as detailed in Supplementary Figure S1A and in M&M section. Briefly, AB wild type zebrafish embryos were exposed to ETU 100 μM (ETU, from now on), CPF 30 nM, and CPF 300 nM according to previous published data [44,45,46,47] that were also experimentally verified by us during the setting up of our models, starting from 6 h post fertilization (hpf) until adulthood (180 dpf). Both pesticides and their doses are environmentally relevant for the areas interested by a massive agricultural activity [63].

**Figure 5:**
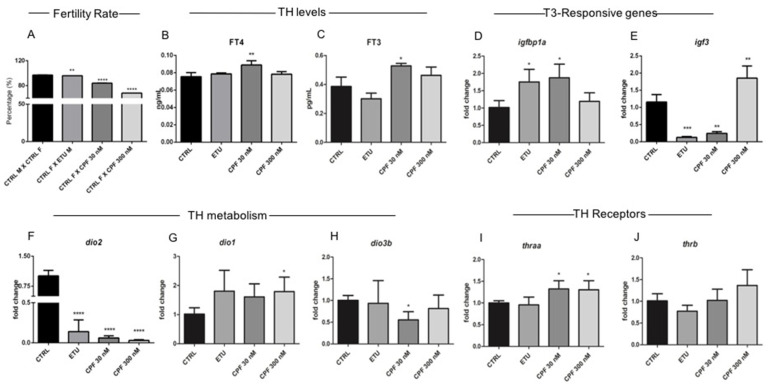
Zebrafish developmentally and long-life exposed to CPF and ETU exhibits male infertility and testicular hyperthyroidism. (**A**) Graph represents the fertilization percentage estimated counting the number of fertilized eggs obtained from three independent matings involving zebrafish males exposed to pesticides vs. females not exposed. (**B**,**C**) FT4 and FT3 levels of zebrafish adult testes (N° = 3 testis/group) measured by ELISA assay, as described in M&M section. RT-qPCR analysis of the mRNA levels of T3 responsive transcripts (*igfbp1a* and *igf3*) (**D**,**E**); of the TH activation and inactivation enzymes (*dio2*, *dio1*, and *dio3b*) (**F**–**H**); of TH receptors (*thraa* and *thrb*) (**I**,**J**). RT-qPCR analyses were performed on three independent experiments from zebrafish adult testes (N° = 3 testis/group). Data analysis was conducted as described in M&M section. Data are reported as fold change values calculated as a ratio between average relative gene expression in treated and control, after normalization on *β-actin* mRNA. Mean and standard deviation are reported. Significant difference from the control group is indicated by * *p* < 0.05, ** *p* < 0.01, *** *p* < 0.001. One-way ANOVA (post hoc test: Dunnett’s) was used.

Breeding behaviour was investigated mating the exposed males with not exposed females, as described in M&M section. Fertilized embryos were obtained from unexposed groups (CTRL, from now on) in all the matings (Figure 5A). However, when treated males were crossed with control female, we observed a reduced ability to produce fertilized eggs. This reduction was more evident in CPF treated males (Figure 5A). Since it is hard to determine the circulating level of fT3 and fT4 in zebrafish, we assessed their levels directly in testes by ELISA (N° = 3 testes/group). As showed in Figure 5B,C, we found a statistically relevant increase of both fT3 and fT4 levels in CPF 30 nM treated males. In ETU and CPF 300 nM testes we observed a fT4 level comparable to control, while a slightly increase of fT3 was evidenced in CPF 300 nM. Then, we assessed the expression of two T3 regulated genes, *igfbp1a* and *igf3* [26], playing a key role in testes development and function. The data revealed an increase of *igfbp1a* mRNA in ETU and CPF 30 nM testes and a down-regulation of *igf3* in the same samples (Figure 5D,E). CPF 300 nM testes showed a normal *igfbp1a* level and *igf3* mRNA upregulation (Figure 5D,E). Considering the altered levels of testicular fT4 and fT3 together with the regulation of T3 responsive transcripts, we analyzed the expression of the deiodinases (*dio1*, *dio2*, and *dio3b*, Figure 5F–H) and TH receptors (*thraa* and *thrb*; Figure 5I,J). The *dio2* gene is the most abundantly expressed deiodinase in zebrafish testis as verified by RT-qPCR (Supplementary Figure S2). Interestingly, we observed a strong reduction of *dio2* mRNA in all the exposed testes vs. control (Figure 5F). *Dio1* transcript was up-regulated only in CPF 300 nM testes and a trend toward the increase was observed in ETU and CPF 30 nM samples (Figure 5G). Noteworthy, Dio1 is a di-functional deiodinase in zebrafish since it mediates both activation and inactivation of THs. Concerning THs inactivation, we observed a decrease of *dio3b* mRNA only in CPF 30 nM (Figure 5H). The analysis of TH receptors expression profile, revealed a transcriptional regulation only for *thraa* gene (Figure 5I,J), with a statistically significant increase in both CPF 30 and 300 nM samples (Figure 5I). Altogether these results indicate that both pesticides induced a hyperthyroid status of testis in zebrafish.

### 3.5. Change in Testis T3 Level Alters Sertoli and Leydig Gene Expression and Estrogen Signalling in Zebrafish Testis

**Figure 6:**
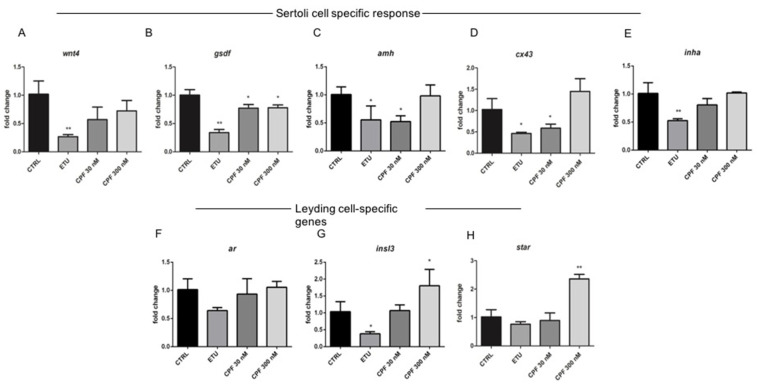
Developmental and life-long exposure to ETU and CPF affects gene expression of Sertoli and Leydig cells in Zebrafish. (**A**–**E**) RT-qPCR analyses of the levels of mRNAs markers of Sertoli cells proliferation and differentiation (*wnt4*, *gsdf*, *amh*, *cx43*, *inha*) and of Leydig cells (*ar*, *insl3*, *star*) (**F**–**H**). RT-qPCR analyses were performed on dissected zebrafish adult testes (N° = 3 testis/group). Data analysis was conducted as described in M&M section. Data are reported as fold change values calculated as a ratio between average relative gene expression in treated and control, after normalization on *β-actin* mRNA. Mean and standard deviation are reported. Significant difference from the control group is indicated by * *p* < 0.05, ** *p* < 0.01. One-way ANOVA (post hoc test: Dunnett’s) was used.

Considering that T3 regulates SC gene expression in adult zebrafish testis, we investigated the levels of selected SC-specific transcripts that modulate the spermatogonial proliferation and differentiation in teleost. We choose *gsdf*, predominantly expressed in SCs and surrounding cells in mature gonads playing an important role in male germ cell proliferation and testicular differentiation [64,65] and *amh.* Both negatively regulate spermatogenesis in the adult teleost testis [32,66]. Moreover, we analyzed *wnt4* gene expression because of its role in differentiation of mammalian SCs. Finally, we tested the level of *inha*, and *cx43* mRNAs, because involved in proliferation and differentiation/maturation of SCs in mammals [60,67]. Noteworthy, some of them, as *cx43*, are T3-regulated transcripts [66]. A relevant dysregulation of several SC specific genes was detected. In particular, we observed the down-regulation of *wnt4* transcript in ETU testes and a trend towards the decrease in CPF 30 nM and 300 nM samples compared with control (Figure 6A). The *gsdf* mRNA showed a strong reduction in all treated testes (Figure 6B), while both *amh* and *cx43* levels were statistically decreased in ETU and CPF 30 nM (Figure 6C,D). We also observed a significant down regulation of *inha* mRNA only in ETU sample (Figure 6E). The analysis of the androgen receptor (*ar)* and *insl3* transcripts, specific markers of LCs, revealed a decreased expression of both in ETU sample, while we found an increase of *insl3* mRNA only in CPF 300 nM testes (Figure 6F,G).

**Figure 7:**
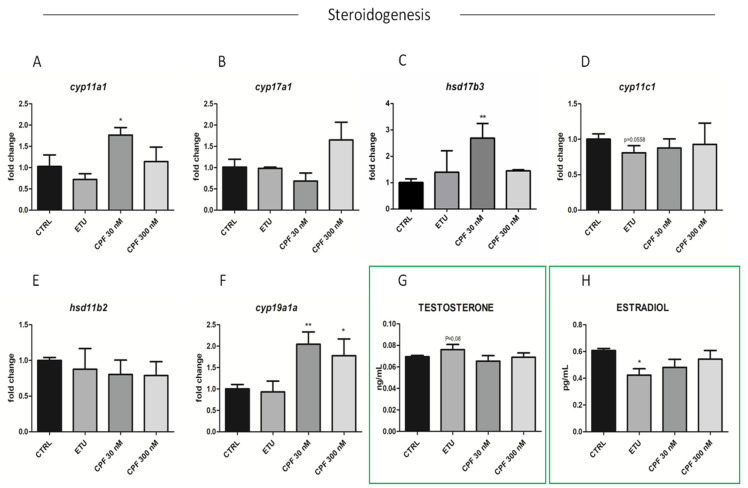

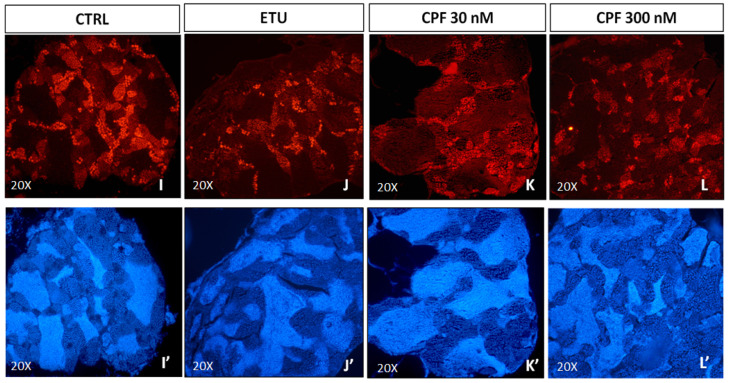
Developmental and long-life exposure to ETU and CPF impairs Steroidogenesis in adult zebrafish. (**A**–**F**) Expression levels of mRNAs of steroidogenic enzymes (*cyp11a1*, *cyp17a1*, *hsd17b3*, *cyp11c1*, *hsd11b2*, *cyp19a1a*). RT-qPCR analyses were performed on zebrafish adult testes (N° = 3 testis/group). Data analysis was conducted as described in M&M section. Data are reported as fold change values calculated as a ratio between average relative gene expression in treated and control, after normalization on *β-actin* mRNA. Mean and standard deviation are reported. Significant difference from the control group is indicated by * *p* < 0.05, ** *p* < 0.01. One-way ANOVA (post hoc test: Dunnett’s) was used. (**G**,**H**) Testosterone and estradiol levels detected by ELISA assay on zebrafish adult exposed vs. CTRL testes (*n* = 3 testis/group). Significant difference from the control group is indicated by * *p* < 0.05. (**I**–**L**) Staining for Erα on zebrafish testis sections (20× magnification), showing the alteration of Erα level in exposed groups (**I’**–**L’**) DAPI staining to visualize nuclei.

Regarding the effects on steroidogenesis, we analyszed the expression levels of key enzymes involved in androgen and estrogen production in zebrafish (Supplementary Figure S3A). Unfortunately, we were not able to determine the main zebrafish androgen, 11- ketotestosterone (11-KT), but we analyzed the levels of free testosterone and E2 in tissue homogenate by ELISA (Figure 7G,H). The level of the former was found increased, whereas the latter was, consequentially, reduced only in ETU sample (Figure 7G,H). Then, we measured the expression of key steroidogenic enzymes in this pathway, including *star.* This last was up-regulated only in CPF 300 nM vs. CTRL testes (Figure 6H). Interestingly, also the expression of *cyp11a1*, *cyp17a1*, *hsd17b3*, and *cyp19a1a* were found up-regulated only in CPF treated groups (Figure 7A–F). None major differential expression was found for the other analyzed transcripts (Figure 7D,E). Finally, we assessed the levels of Erα protein in testes from exposed zebrafish by immunohistochemistry assay (IHC, Figure 7I–L), staining nuclei with DAPI (Figure 7I’–L’). Although not quantitative, the IHC data evidenced a reduction of Erα in CPF exposed testes (Figure 7K,L). These data suggest that both pesticides, besides altering the testicular T3 signalling, influence the maturation, differentiation and activity of SCs, and regulate gonadal steroidogenesis also in zebrafish.

### 3.6. Examination of Treated Testes and Analysis of Key Spermatogenesis Genes in Zebrafish

**Figure 8:**
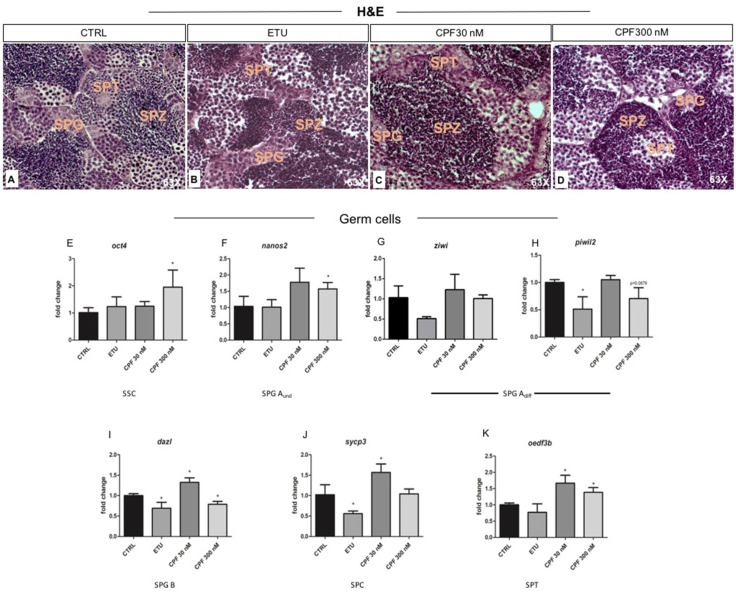
Analysis of testis morphology and molecular markers of spermatogenesis in zebrafish developmentally and long life exposed to CPF and ETU. (**A**–**D**) Hematoxylin and Eosin staining of zebrafish testes sections from adult animals exposed to pesticides and not exposed (CTRL) (63× magnification). The different germ cells sub-population are evidenced. RT-qPCR analyses of the expression profiles of different germ cells markers: starting from stem cell/undifferentiated spermatogonia (*oct4*, *nanos2*) (**E**,**F**), differentiated spermatogonia type A and B (*ziwi*, *piwil2*, *dazl*) (**G**–**I**), and differentiated spermatocytes and spermatids (*sycp3*, *oedf3b*) (**J**,**K**). RT-qPCR analyses were performed on dissected zebrafish adult testes (N° = 3 testis/group). Data analysis was conducted as described in M&M section. Data are reported as fold change values calculated as a ratio between average relative gene expression in treated and control, after normalization on *β-actin* mRNA. Mean and standard deviation are reported. Significant difference from the control group is indicated by * *p* < 0.05. One-way ANOVA (post hoc test: Dunnett’s) was used. SSC = spermatogonial stem cell; SPG = spermatogonia; SPG A_und_ = undifferentiated spermatogonia type A; SPG A_diff_ = differentiated spermatogonia type A; SPG B = spermatogonia type B; SPC = spermatocyte; SPT = spermatids; SPZ = spermatozoa.

Since THs influence maturation and activity of testicular somatic and germ cells, we analyzed testicular morphology by using histology of testicular sections. Spermatogonia (SPG), spermatocytes (SPC), and spermatids (SPT) were present and evidenced in CTRL testes (Figure 8A). In both ETU and CPF treated zebrafish testes, the normal testis cyto-architecture was present, even if it appeared disorganized in terms of fraction occupied by the different sub-population of germ cells (Figure 8B–D). A higher volume fraction of differentiated germ cells was observed in both CPF treated testes (Figure 8C,D), while in ETU was the opposite (Figure 8B). This observation was molecularly supported by the RT-qPCR analysis of the transcripts marking the germ cells at different stages. Interestingly, we found an up-regulation of *oct4* mRNA, marker of stem cell spermatogonia, and nanos homolog 2 (*nanos2*) mRNA, marker of A_und_ spermatogonia, in CPF 300 nM treated sample (Figure 8E,F). Normal level of piwi-like RNA-mediated gene silencing 1 (*piwil1*, named *ziwi*), marking all type A spermatogonia, and a decreased expression *dazl*, marker of type B spermatogonia (Figure 8G,I) were retrieved in CPF 300 nM testes. None change was observed in spermatocyte marker synaptonemal complex protein 3 (*sycp3*) (Figure 8J). The spermatid marker outer dense fiber of sperm tails 3B (*oedf3b*) mRNA was induced in CPF 300 nM (Figure 8K). In CPF 30 nM samples, we observed an up-regulation of mRNAs specifically expressed in type B spermatogonia (*dazl*) and in differentiated germ cells (*sycp3* and *oedf3b*) (Figure 8I–K). In ETU we found a down regulation of all markers of differentiated germ cells (*ziwi*, *piwil2*, *dazl*, *sycp3*), except for *oct4*, *nanos2*, and *oedf3b* transcript (Figure 8E–K). Altogether, these results indicate the existence of a treatment-specific impairment of spermatogenesis, with ETU associated to a reduction of spermatogonial differentiation and CPF exerting an opposite effect.

## 4. Discussion

Thyroid hormones are important regulators of testis functionality that might be impaired by parental and developmental exposures to CPF and ETU [41,43,60]. Besides their thyrotoxic activity [37], recent work of our laboratory has suggested that peripheral imbalance of T3 metabolism and signalling could be also a mechanism of action of both pesticides in zebrafish liver [39]. Thus, we checked if t-T3 metabolism and signalling could be an evolutionary-conserved target of both pesticides, hopefully applicable to humans. We analyzed the effects of the exposure, in vivo, in evolutionary distant vertebrates and in ex-vivo cultures of murine seminiferous tubules trying to reproduce in terms of dose and exposure window a scenario relevant to humans. Although in different experimental settings, previous results evidence that CPF and ETU exposure may cause degeneration of seminiferous tubules, exert a negative effect on sexual hormones levels, alter the expression of steroidogenic enzymes, and, finally, impair sperm production [68,69,70]. The here reported alteration of serum testosterone level and of steroidogenic enzymes expression (Figure 1B,C) agrees with previously published data [68,69,70]. Vice versa, the effect at CPF-H treatment is not in line with them. Despite the trend toward the increase in all exposed-groups of serum levels of E2 showed, particularly evident in ETU-H group (Figure 1A) and of *Cyp19a1* mRNA (Figure 1D), the analysis of testicular CYP19A1 protein reveal a decreased expression in both ETU and CPF treated groups, with the only exception for the lower dose of ETU (Figure 1G,G’,I,I’). This might depend by post-translational regulation of protein level. Recently, it has been demonstrated that CYP19A1 can be post-transcriptionally regulated to modulate spermiogenesis completion [71] and that different miRNAs might modulate its levels also in testes. Among them, miRNA 378 is expressed in SC and it is regulated by different EDCs [72,73]. ERα mRNA and protein were found altered only in CPF- treated mice but not in ex-vivo cultured seminiferous tubules (Figure 1E,H,H’). These last results are contrasting with previous published in vitro data evidencing none major effects of the exposure, although different cells were adopted [74]. Overall, the discrepancy among the data might depend on the adoption of different exposure models in terms of dosage, duration, and used animals or cells [41] evidencing the need to harmonize the exposure settings to draw conclusion. Furthermore, this discrepancy suggest that CPF might regulate ERalpha expression by different mechanisms and a more comprehensive approach will be needed for their identification and investigation of their interplay.

Interestingly for us, T3 signalling can inhibits mRNA expression of estradiol receptor, and can directly act on mRNA levels of steroidogenic enzymes and steroidogenic acute regulatory protein [75]. Despite none major difference is detected in the testicular level of T3, a reduced testicular T3 signalling is evidenced in the exposed mice, by mean of the levels of T3-responsive transcripts as *Spot14*. However, *deiodinase 2* expression was reduced in almost all the exposed groups. *Deiodinase 3* mRNA was increased only in ETU-L and CPF-H males even if DIO3 protein was found augmented in all the exposed testes except ETU-H (Figure 2H,I). This was surprising since its degradation via proteasome pathway has not been described and it has a long half-life 3, so other mechanisms might be involved [76]. Noteworthy, the data point to a testicular hyperthyroidism that is also supported by the trend towards the increase of *Cx43* mRNA (Figure 4C), a T3-responsive gene in testis, detected despite the reduced level of *Thra* transcript (Figure 2F). These contrasting results might be explained by the complex organization of the promoters of the investigated genes. As example, *Cx43* mRNA is also a target of FSH signalling in murine testes [77] whereas *Spot14* (Figure 2G) is transcriptionally regulated also by SREBP-1 and ChREBP pathways [78,79,80,81], implying again that other signalling pathways might be targeted by these pesticides together with T3 signalling. The discussed above evidence show difficulties to verify in vivo if the deregulation of E2 metabolism and signalling is directly promoted by pesticides or by their activity on TH signalling. The experiments conducted in an ex-vivo mouse seminiferous tubule culture model, using a culture media deprived of steroids hormones, demonstrate the direct effect of both compounds. In particular, our results confirm T3 as a positive modulator of estrogen synthesis and signalling in testis and highlight that both compounds act as regulator of the expression of genes involved in T3 metabolism. T3 co-exposure not always slightly lowered their effects, confirming that different mechanisms can be involved up to the promoter organization of each gene. As example, *Spot14* mRNA level is not affected by pesticides but less efficiently induced by T3 when pesticides are in the culture media. This suggests that they might act directly or also antagonizing the T3 activity. This observation, although at less extent, is confirmed also for E2 metabolism and signalling. Indeed, T3 co-administration lowered the effect of ETU suggesting that testicular T3 modulation plays a role in protecting testicular health.

T3 is likely to represent a major hormonal signal involved in the establishment of the adult SC population. Transient juvenile hyperthyroidism resulted in an early cessation of SC proliferation and had a concomitant stimulatory effect on their maturation [6]. In our analysis, we observe a decrease of SC proliferation markers (as *Inha*) and an increase of SC differentiation marker (as *Cx43*, Figure 4A–E) that is in line with the literature [62]. *Cx43* decrease prevents initiation of spermatogenesis and leads to a significant reduction of germ cells and infertility [82,83]. Indeed, an increase expression of marker of stem cell/undifferentiated spermatogonia, marked by *Oct4* (Figure 4F) was detected. On the contrary, *Stra8*, marker of differentiated spermatogonia, showed a decrease in all the exposed groups (Figure 4G). Unfortunately, we were not able to investigate the tissue morphology in mouse samples at the time of our analysis to better evaluate the effects on germ cells population, but our results seem to be in accordance to recently published data [84]. Surely, mechanisms other than disarrangement of testicular T3 metabolism and signalling may intervene in the regulation of spermatogenesis in mouse testes exposed to CPF and ETU. However, the reported results suggest that chronic exposure of mice to CPF and ETU started during the foetal life, supported also by the in vitro data, alters the testicular T3 homeostasis. The data suggest its damage as part of the mechanisms responsible of the damage of the testicular function, acting directly on germ and somatic cells.

Interestingly, we describe similar effects in zebrafish exposed to low doses of CPF and ETU. Indeed, also in this model, evolutionary distant, we observed the presence of THs imbalance following chronic exposure to both pesticides (Figure 5B–J), mainly leading to testicular imbalance of T3 homeostasis in particular in the CPF 30 nM-exposure group. However, the *cx43* mRNA was found reduced in treated samples (Figure 6D) suggesting a possible difference between mouse and zebrafish. This may involve the modulation of gonadotropins or depend on stimulatory role of T3 on SC proliferation already observed in adult zebrafish testis [24]. Indeed, the effects of T3 in adult teleost testes has been recently studied and clarified better than in mice [24,26,29]. As defined by these works, T3 stimulates FSH-responsive *Igf3*, a SC growth factor exclusively expressed in zebrafish gonads [80] and mediates the increase of some *igfbps*, such as *igfbp1a* transcripts [26,29]. The inhibition of *igf3* observed in ETU and CPF 30nM despite the increase of T3, may indicate a negative impact on FSH synthesis by pituitary. Since T3 induces the proliferation of type A undifferentiated spermatogonia (A_und_) and of SCs in zebrafish testis via SC-derived *igf3*, [26], it is possible that the increase in *igfbp1a* modulates spermatogonial proliferation and differentiation behaviour, particularly in a condition of T3 excess. Indeed, Safian et al. (2016) showed a depletion of type A_und_ spermatogonia in zebrafish testis treated with T3 when neutralizing the *Igfbps*. This suggests that Igfbps protect undifferentiated spermatogonia against excessive differentiation. Interestingly, our analysis showed the increase of *oct4* and *nanos2* mRNAs, markers of stem cells spermatogonia (SSC) and spermatogonia A_und_ respectively, in CPF 300 nM treated testes (Figure 8E,F) in which we found a normal level of *igfbp1a* and an increase of *igf3* transcript. Vice versa, in ETU and CPF 30 nM, in which *igfbp1a* expression was increased, we recorded a normal level of SSC and SPG A_und_ markers (Figure 8E,F). However, the expression of differentiated germ cells markers was reduced in ETU samples and inversely regulated in CPF 30 nM treated testes (Figure 8G–K). The reported difference in the effects on spermatogenesis between ETU and CPF 30 nM may depend by other mechanisms. Indeed, insulin-like 3, *insl3*, encoding spermatogenic signalling molecules expressed by LCs and required only for proliferation and differentiation of A_und_ spermatogonia, was downregulated only in ETU (Figure 6G). Furthermore, the decreased availability of *igf3* suggests a compromised function of SCs corroborated by the reduction of all SC markers analyzed in both CPF 30 nM and ETU (Figure 6A–E). Probably, the decrease of *amh* transcript in CPF 30 nM induces the recruitment of germ cells into differentiation phase, usually observed in teleost testis [85]. Finally, in agreement with previous reports showing that T3 did not induce major changes in androgen levels in fish, the data evidenced only a slight increased level of testosterone in ETU-treated testes [86, that might potentiate Fsh-effects on steroid release and gene expression. Further works need to better clarify this hypothesis.

## 5. Conclusions

In our multi-species analyses, conducted in vivo and in vitro, on the mechanisms of testicular toxicity exerted by developmental and long-life exposure to CPF and ETU in both models, we evidenced the local customization of T3 signalling as a common a primary target of both compounds. In all the adopted models the result of the exposure was the damage of testicular T3 homeostasis and we evidence that pesticides can also act antagonizing T3 activity. The in vitro data suggest that the impairment of testicular homeostasis is part of more complex mechanisms involving also other pathways. They alter also of somatic and germ cell proliferation and differentiation. Regarding these last, the spermatogonia compartment seems to be mostly damaged in both models. These effects are even more evident in zebrafish leading to define it as a good model to investigate the role of t-T3 metabolism and signalling at the adulthood together with the ex-vivo cultures of seminiferous tubules. These lasts will allow the multiple testing reducing the number of the enrolled animals. Since, more studies are needed to better deciphering t-T3 signalling as target of testicular toxicity, the zebrafish model give an opportunity to look at single cell level since the feasibility of these approach has been tested for other organs and disease.

## Data Availability

Not applicable.

## Supplementary Materials

The following are available online at https://www.mdpi.com/article/10.3390/cells10092187/s1, Table S1: List of primers used for RT-qPCR. Figure S1: CPF and ETU treatment timeline in mouse and zebrafish models; Figure S2: CT values of zebrafish and mouse testis deiodinases; Figure S3: Steroid biosynthesis pathway in zebrafish.
