# Supplementary material for: Multi Species Analyses Reveal Testicular T3 Metabolism and Signalling as a Target of Environmental Pesticides
Source: Cells. 2021 Aug 25;10(9):2187. doi: 10.3390/cells10092187 (PMC8471965; doi:10.3390/cells10092187)
Supplement: Supplementary file 1 [file cells-10-02187-s001.zip › cells-1248765-supplementary.pdf]

Supplementary Table S1. List of primers used for RTqPCR analysis

Gene Name	Gene symbol		Primers sequence (5'-3') Foward	Reverse
Beta-actin	*Bactin*	Mouse	GTCGAGTCGCGTCCACC	GTCATCCATGGCGAACTGGT
		Zebrafish	TCTCTTAAGTCGACAACCCCC	TCTGAGCCTCATCACCAACG
Steroidogenic acute regulatory protein	*StAr*	Mouse	CAGGGAGAGGTGGCTATGCA	CCGTGTCTTTTCCAATCCTCTG
		Zebrafish	ATGCCTGAGCAGAAGGGATTT	CCACCTGGGTTTGTGAAAGT
Cytochrome P450 aromatase	*Cyp19a1*	Mouse	CATGGTCCCGCAAACTGTGA	GTAGTAGTTGCAGGCACTTC
		Zebrafish	TCAGACACCATCGACCACAC	TCCCAGACCAGATGAACCGA
Estrogen Receptor Alpha	*Er-α*	Mouse	GGCTACCATTATGGGGTCTGG	CCCACTTCGTAACACTTGCG
Deiodinase 1	*Dio1*	Mouse	CCACCTTCTTCAGCATCC	AGTCATCTACGAGTCTCTTG
		Zebrafish	GTTCAAACAGCTTGTCAAGGACT	AGCAAGCCTCTCCTCCAAGTT
Deiodinase 2	*Dio2*	Mouse	CCTCTTCCTGGCGCTCTATG	TTCAGGATTGGAGACGTGCA
		Zebrafish	TTCTCCTTGCCTCCTCAGTG	AGCCACCTCCGAACATCTTT
Deiodinase 3	*Dio3*	Mouse	CCGACCTGATGGCTTCCA	CGCGCCATGAACGGTGGTCA
		Zebrafish (*dio3b*)	TCCTGACCGCCCTTCATGAC	TGCGCCTCCTCGATGTACAC
Thyroid Hormone receptor Alpha	*Thra*	Mouse	GCCATTGGAAACAGAGGCGA	ATCTGGTCTTCGCAAGGCA
		Zebrafish (*thraa*)	CAATGTACCATTTCGCGTTG	GCTCCTGCTCTGTGTTTTCC
Thyroid hormone receptor Beta	*thrb*	Zebrafish	CGCAGTTTGGCAGGTAGAAT	ATAGGTGCCGATCCAATGTC
Thyroid hormone responsive protein	*Spot14*	Mouse	ATGCAAGTGCTAACGAAACGC	CCTGCCATTCCTCCCTTGG
Anti-Müllerian	*Amh*	Mouse	GGCTAGGGGAGACTGGAGAA	AGGTGGAGGCTCTTGGAACT

Hormone		Zebrafish	ACAGTGAGGCACGAAGAGCAG	TGGCATATTGGTCAGTTGGCTGT
SRY-box transcription factor 9	*Sox9*	Mouse	TCTGGAGGCTGCTGAACGAG	GCTTGTCCGTTCTTCACCGA
Wnt Family Member 4	*Wnt4*	Mouse	CGAGCAATTGGCTGTACCTG	TCCGGAACTGGTATTGGCAC
		Zebrafish	GCGTTCCTGTGTTTGGGAAA	TCCTGACCACTGGAAACCTTCT
Inhibin Subunit Alpha	*Inha*	Mouse	TGAACCAGAGGAGGAAGATGTCTC	TCTGGCAGCTGGCTGGTC
		Zebrafish	TCTGTTCCCAAGCTCAGAGTC	TCCAGCATCAGAAGAGTGGC
Gonadal somatic cell derived factor	*gsdf*	Zebrafish	GGTGTTGATGAAAGATCTGGGC	ATGTAGACGAACGGCACGAT
POU class 5 homeobox 1	*Oct4*	Mouse	GCCAGACCACCATCTGTCGCT	AGGGTCTCCGATTTGCATATCTCCT
		Zebrafish	ACGCAGGCAGATGTGGGACTC	AGGGTTCTCGGAGTTTTCGGC
Connexin 43	*Cx43*	Mouse	ACAGCGGTTGAGTCAGCTTG	GAGAGATGGGGAAGGACTTGT
		Zebrafish	CACGCCGAAGGAACTGTCTA	GCAAGAGTTGGTCCTTTCGC
Stimulated by retinoic acid gene 8	*Stra8*	Mouse	ACCGTGGTGGCCTTAAAGATTA	TGAAGAGCCCTACCAGGGTG
Synaptonemal complex protein 1	*Sycp1*	Mouse	GGTTCCGTTCCATGTCGTCT	TGGCAGTCTTGAAGTAGTTGGA
Nanos homolog 2	*nanos2*	Zebrafish	ACTAAAGGCAAGGGACGGAC	GTTTCTCCGCGGACAGTAGT
Piwi-like RNA-mediated gene silencing 1	*ziwi*	Zebrafish	AGTCACTCGTCCAGAGTGGT	CTGGTTTAAGGCCGCTGTTG
Piwi-like RNA-mediated gene silencing 2	*piwil2*	Zebrafish	TGATACCAGCAAGAAGAGCAGATCT	ATTTGGAAGGTCACCCTGGAGTA
Deleted in azoospermia-like	*dazl*	Zebrafish	ACTGGGACCTGCAATCATGA	AATACAGGTGATGGTGGGGC
Synaptonemal complex protein 3	*sycp3*	Zebrafish	CGGATCTGACGAAGACACGA	TGAACCTCATTCCCCACACC

outer dense fiber of sperm tails 3B	*oedf3b*	Zebrafish	ATGACCAAACTCCAGGTCCAG	AGACGTTTGGGGCAGATGTT
insulin-like 3	*insl3*	Zebrafish	ACTTCGCATACCCTTATAGGAATCT	ACGAGGTCTCTATCCAGCCA
Androgen receptor	*ar*	Zebrafish	ATGGGCCAAAGGACTTCCAG	AGACGTGCATCCTACGATCATT
insulin-like growth factor binding protein 1a	*igfbp1a*	Zebrafish	AACGCGATACGCAAGAAACT	CGCAGTTTGGCAGGTAGAAT
insulin-like growth factor 3	*igf3*	Zebrafish	TGTGCGGAGACAGAGGCTTT	CGCCGCACTTTCTTGGATT
cytochrome P450, family 11, subfamily A, polypeptide 1	*cyp11a1*	Zebrafish	TATCACGTCTCCACAGGCGG	CTGTTCCTCCATCGTCCAGG
cytochrome P450, family 17, subfamily A, polypeptide 1	*cyp17a1a*	Zebrafish	GACCTGTTTCTCCGCTCCTC	AAGCGTCCTGGGTCAAAGAG
hydroxysteroid (17-beta) dehydrogenase 3	*hsd17b3*	Zebrafish	CTTGCGCCATCCTGGTTTTT	TTCGAAAGCTGCCCATTTCC
cytochrome P450, family 11, subfamily C, polypeptide 1<br>*cytochrome P450, family 11, subfamily C, polypeptide 1*	*cyp11c1*	Zebrafish	AAGACGCTCCAGTGCTGTG	CCTCTGACCCTGTGATCTGC
hydroxysteroid (11-beta) dehydrogenase 2	*hsd11b2*	Zebrafish	TTATCAACACACTTCGTCAC	TCTCCAGCAGATATTCTTCC

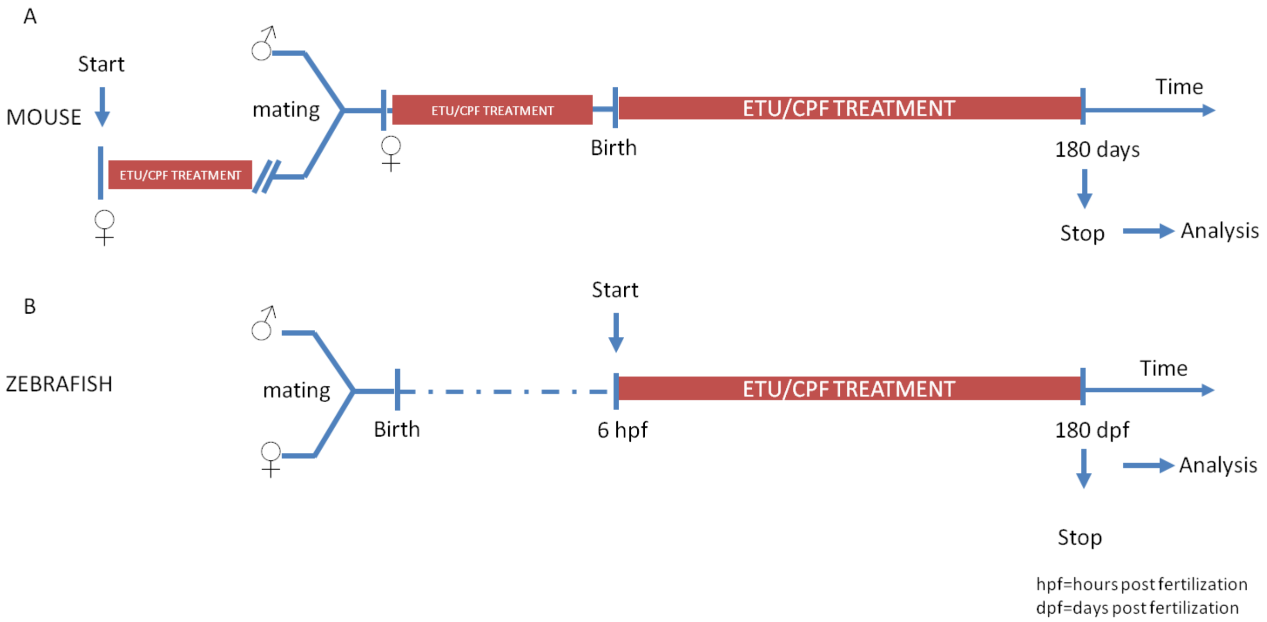

Supplementary Figure S1. CPF and ETU treatment timeline in mouse and zebrafish models. (A) Treatment timeline in mouse. Seven days before mating, female mice were exposed to ETU and CPF. The treatment of the mice enrolled in the studies was continued after the conception and at birth until the adulthood (180 days) by direct feeding. Details concerning mouse background and pesticides concentration are described in Material and methods section (M&M section, from now on). (B) Treatment timeline in zebrafish animals. Embryos from wild type parents were collected and blastulae were selected. At 6 hours post fertilization (hpf) embryos were randomly assigned to experimental groups and treated until the adulthood (180 days). Detailed protocols are reported in M&M section.

ZEBRAFISH		
SAMPLE	GENE	CT
CTRL 1	*dio1*	26,361676
CTRL 2	*dio1*	25,8132513
CTRL 3	*dio1*	26,1792207
CTRL 1	*dio2*	23,9636907
CTRL 2	*dio2*	23,568608
CTRL 3	*dio2*	23,704325
CTRL 1	*dio3b*	28,2842573
CTRL 2	*dio3b*	27,883634
CTRL 3	*dio3b*	28,5107633
CTRL 1	*dio3a*	31,6899675
CTRL 2	*dio3a*	31,35423
CTRL 3	*dio3a*	30,647417

MOUSE		
SAMPLE	GENE	CT
CTRL 1	*Dio1*	32,5564197
CTRL 2	*Dio1*	32,5280177
CTRL 3	*Dio1*	32,8509027
CTRL 1	*Dio2*	25,4866547
CTRL 2	*Dio2*	24,9941063
CTRL 3	*Dio2*	26,502855
CTRL 1	*Dio3*	32,4084823
CTRL 2	*Dio3*	32,7829523
CTRL 3	*Dio3*	33,3936747

Supplementary Figure S2. CT values of zebrafish and mouse testis deiodinases. RTqPCR analyses were conducted to determine the deiodinases mainly expressed in zebrafish and mouse testes. CT means of each single biological replicate (N=3 testis/group) are shown. The CT values represent TH enzymes of synthesis (zebrafish, *dio2;* mouse *Dio1, Dio2*) and degradation (zebrafish *dio*, *dio3b* and *dio3a;* mouse *Dio3*) genes in control testes, obtained in independent experiment using RTqPCR assay.

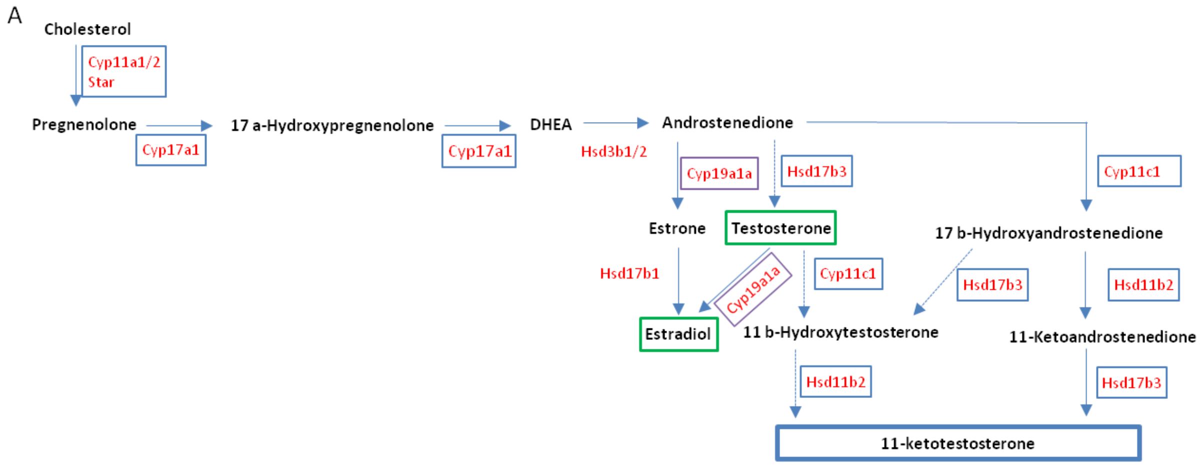

Supplementary Figure S3. Steroid biosynthesis pathway in zebrafish. (A) Schematic representation of pathways for production of sex steroids in zebrafish. The principal pathway for 11-Ketotestosterone (11-KT) biosynthesis is indicated with solid arrows, whereas minor alternative pathways are indicated with dashed arrows. In green boxes are inserted estradiol and testosterone, while blue boxes are defined the involved enzymes in the pathways. DHEA, dehydroepiandrosterone. Adapted from James et al., 2019

References

Oakes JA, Li N, Wistow B.C.R, Griffin A. et al., Ferredoxin 1b Deficiency Leads to Testis Disorganization, Impaired Spermatogenesis, and Feminization in Zebrafish. Endocrinology, 2019, 160(10):2401–2416. doi: 10.1210/en.2019-00068
